# Nanoscale Ferroelectric Programming of van der Waals Heterostructures

**DOI:** 10.1021/acs.nanolett.4c03574

**Published:** 2024-12-13

**Authors:** Dengyu Yang, Qingrui Cao, Erin Akyuz, John Hayden, Josh Nordlander, Ian Mercer, Muqing Yu, Ranjani Ramachandran, Patrick Irvin, Jon-Paul Maria, Benjamin M. Hunt, Jeremy Levy

**Affiliations:** † Department of Physics, Carnegie Mellon University, Pittsburgh, Pennsylvania 15213, United States; ‡ Department of Physics and Astronomy, University of Pittsburgh, Pittsburgh, Pennsylvania 15260, United States; ¶ Pittsburgh Quantum Institute, Pittsburgh, Pennsylvania 15260, United States; § Department of Materials Science and Engineering, The Pennsylvania State University, University Park, Pennsylvania 16802, United States

**Keywords:** ferroelectric, heterostructures, van der Waals, nanoscale potentials

## Abstract

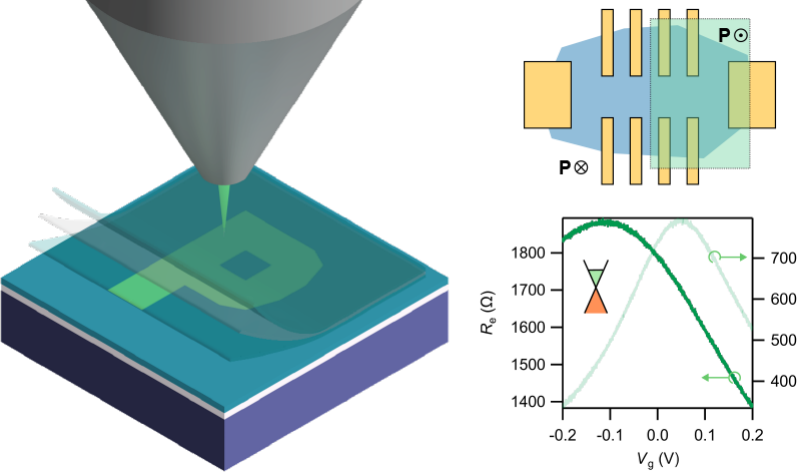

We demonstrate an approach to creating nanoscale potentials in van der Waals layers integrated with a buried programmable ferroelectric layer. Using ultra-low-voltage electron beam lithography (ULV-EBL), we can program the ferroelectric polarization in Al_1–*x*_B_*x*_N (AlBN) thin films, generating structures with sizes as small as 35 nm. We demonstrate the ferroelectric field effect with a graphene/vdW stack on AlBN by creating a p–n junction. This resist-free, high-resolution, contactless patterning method offers a new pathway to integrate ferroelectric films with a wide range of two-dimensional layers including transition-metal dichalcogenides (TMD), enabling arbitrary programming and top-down creation of multifunctional devices.

Van der Waals (vdW) heterostructures have gained significant attention due to their distinctive and diverse properties in low-dimensional materials.^1,2^ Through tuning of various external parameters, efforts to configure and understand vdW quantum materials encompass superconductivity,^3,4^ Mott-like insulator states,^5,6^ ferromagnetism at integer^7−9^ and fractional^10−12^ fillings of the moiré bands, interlayer moiré excitons,^13−16^ and more. To perform an enhanced comprehension and exploration of these properties, as well as for the fabrication of diverse nanoscale quantum devices, the importance of electrostatic manipulation with efficiency and low disorder cannot be overstated. However, achieving such precise and localized control poses substantial challenges, underscoring a critical area for research and technique development.

Most top-down approaches to electrostatic gating of vdW layers rely on electron-beam lithography (EBL) to create nanoscale patterns. This approach has been used to produce electrostatically gated sharp edge states,^17,18^ sharp p–n junctions,^19^ and gating through patterned dielectric substrates.^20−23^ A method utilizing a synergistic approach with an electron beam and backgate demonstrates patterning of vdW materials with 200 nm resolution.^24^ In addition to EBL methods, atomic force microscopy (AFM) has been used for precise patterning with approximately 50 nm resolution, and open-faced vdW materials can be programmed using atomic force microscopy.^25−27^

Ferroelectric materials are appealing to modern nanoelectronics due to their nonvolatile and switchable electric polarization. Recent reports show that solid solutions in the AlN-ScN,^28,29^ AlN-BN,^30−32^ and ZnO-MgO^33^ families support ferroelectricity with polarization values between 80 and 150 μC/cm^2^, with excellent polarization retention and stability, to thicknesses at or below 10 nm. These materials can be processed under conditions that are more chemically and thermally compatible with many mainstream semiconductor platforms.^28−31,33^ As such, they present new possibilities for integration with 2D materials, where programmed polarization patterns can tune electron transport. There are successful achievements regarding using a programmed ferroelectric substrate to tune the 2D materials, including using prepatterned ferroelectric domains^34,35^ and scanning probe patterned ferroelectric domains.^36,37^

Here, we describe an approach to programming ferroelectric thin films that are buried below a thick (>30 nm), multilayer vdW stack. The approach is based on a technique first developed by Nutt et al.^38^ that utilized focused electron beams to switch ferroelectric polarization in LiNbO_3_. We show that by carefully tuning the electron acceleration voltage, we can program the buried ferroelectric Al_1–*x*_B_*x*_N (AlBN) thin film with optimized spatial resolution particularly when using ultra-low-voltage electron beams.^1^ The acceleration voltage (*V*_acc_) is optimized based on Monte Carlo simulations so that it is sufficient to penetrate through the ferroelectric film and minimize unwanted backscattered electrons that can broaden the resolution. Our investigation also explores the impact of electron dose (*D*), and we successfully demonstrate beam conditions that produce feature sizes as small as 35 nm, dimensions that are comparable to the limit of our AFM AC scans and piezoelectric force microscopy (PFM) scans that measure them. We employ this method on the graphene vdW stack on AlBN and demonstrate graphene doping under selectively patterned polarization difference and show a p–n junction as a benchmark device. The method offers a new approach to integrate the ferroelectric film with other materials such as transition metal dichalcogenides (TMDs). It is resist-free processing and has potential for high-resolution patterning without physical contact. This new compatibility is aligned with the increasing complexity of current device concepts that can leverage intimate integration between ferroelectric material thin films, complex oxides, semiconductors, and vdW materials to achieve multifunctional devices.

Al_1–*x*_B_*x*_N (*x* = 0.07) thin films with a target thickness of 11 or 20 nm are grown by dual-cathode reactive magnetron sputtering on W (40 nm)-coated *c*-axis Al_2_O_3_ substrates at 300 °C. It is confirmed from the X-ray diffraction (XRD) measurement that these AlBN have a polycrystalline texture with a strong *c*-axis orientation^31^ (see Supporting InformationFigure S7). These films exhibit robust ferroelectric behavior with a switchable polarization as large as 130 μC/cm^2^, which leads to a surface charge density close to 10^15^ cm^–2^.^30−32^

The sputtering conditions and surface preparations of AlBN growth produce films with a uniform polarization-down dipole orientation and a compensating negative surface charge mechanism.^30−32^ As Figure 1 shows, a letter “P” is polarization patterned onto the ferroelectric AlBN film using the ULV-EBL exposure to switch the dipole moment from as-grown polarization down to polarization up (Figure 1(b–d)). The exposure area dose is 4050 μC/cm^2^. We use CASINO Monte Carlo simulation^39−41^ to simulate the injected electron trajectories ((Figures 1(e) and S1) and determine the optimal electron acceleration voltage using *V*_acc_ to energize the electrons. To penetrate most of the thickness of the AlBN film, a *V*_acc_ = 500 V (1 kV) is used to expose the 11 nm (20 nm) AlBN film. In both cases, the electron energy is sufficient enough to switch the surface polarization and be confirmed under AFM and PFM. A higher acceleration voltage is needed when there is a vdW stack on top of AlBN. As Figure 1(e) shows, a 2 kV *V*_acc_ can penetrate through a graphene/10 nm hexagonal boron nitride (hBN) stack on top and reach AlBN. The polarization difference on the ferroelectric sample with different surface charges is spatially characterized using the AFM AC mode or PFM. The AFM AC mode scan shows signal contrast from the surface charging difference (Figure 1(f)). The PFM scan further shows the contrast between the two different polarized regions (Figure S3). As shown by Calderon et al., the switching mechanism occurs through a sequential inhomogeneous path of localized polyhedral distortions.^42^ These give rise to a local nonpolar transition phase that mediates the global transition.

**Figure 1:**
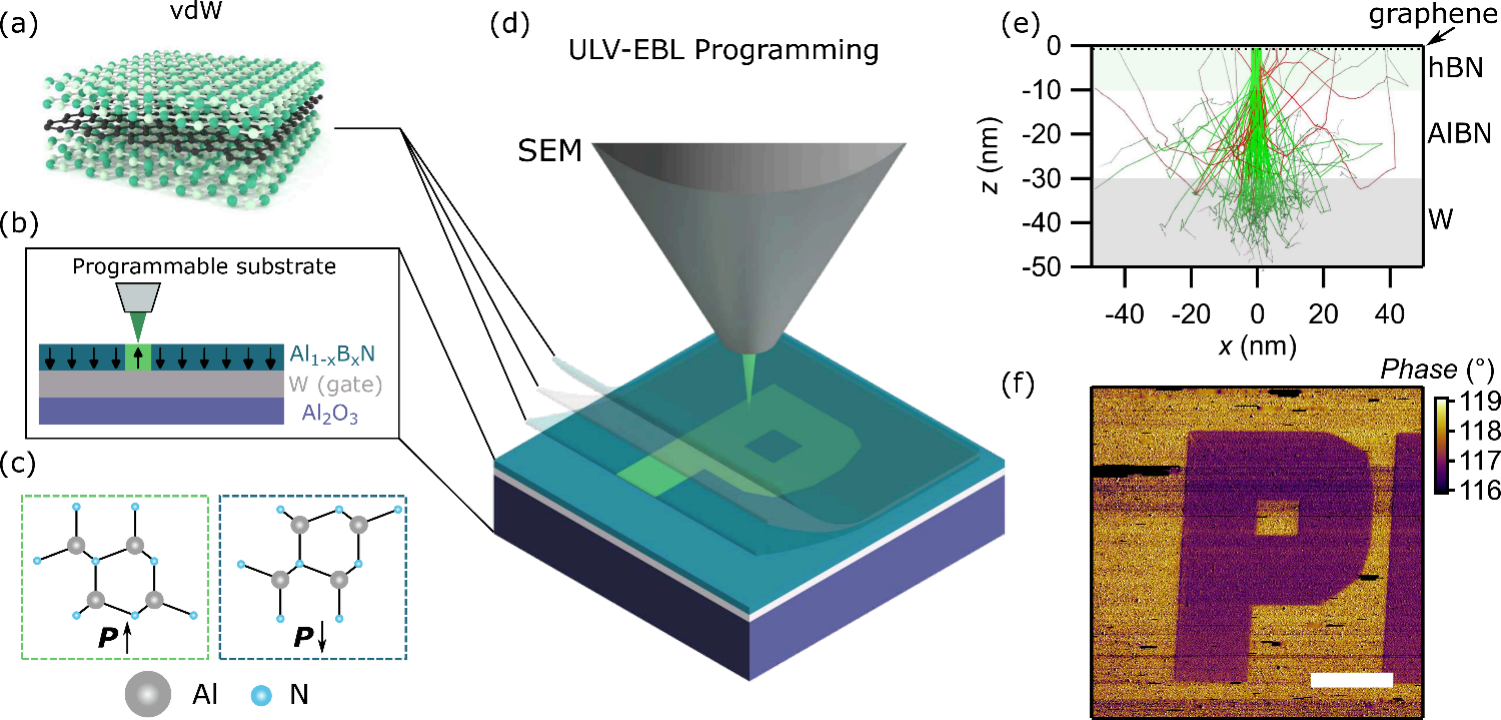
Ferroelectric switching using ULV-EBL. (a) Illustration of the vdW stack (hBN/graphene/hBN). (b) ULV-EBL programming of ferroelectric domains in AlBN grown on W/Al_2_O_3_. (c) Atomic structure of the wurtzite Al(B)N of N-polar and Al-polar cases. (d) Illustration of ferroelectric polarization patterning with ULV-EBL, through the vdW layer. (e) Monte Carlo simulation for electron trajectories of electron beam acceleration voltage *V*_acc_ = 2 kV at a graphene/hBN (10 nm) stack on 20 nm AlBN film. (f) AFM AC phase image of the ULV-EBL-exposed AlBN with a letter “P”. The scale bar represents 4 μm.

While the AFM and PFM results strongly suggest polarization reversal, it is prudent to provide additional support given the companion artifacts that might occur using scanning probe microscopy (SPM)-based measurements, especially for such thin layers where leakage currents can be large. Positive-up negative-down (PUND) measurements^43^ are widely regarded as a gold standard for separating ferroelectric polarization and leakage or dielectric contributions to switching. First we evaluate reference AlBN capacitors prepared from the identical 20 nm parent films (Figure 2(a,b) and Figure S8). We apply a +17 V voltage pulse to the bottom electrode of a 20 nm thick AlBN ferroelectric sample, as Figure 2(a) shows. Since the sample polarization initially points down, the first pulse (P) switches the polarization to up. An identical follow-up pulse (U) is then applied which should not switch the polarization. The measured current from U originates from only dielectric displacement and leakage currents. Thus, the difference between the measured current from P and U pulses shows the current that purely comes from the polarization switching. The identical test is carried for the negative side (N and D) except that the voltage sign is now the opposite. Figure 2(a) shows the current versus time traces for all PUND pulses. For the P and N pulses there are two features, a sharp spike associated primarily with polarization reversal and a decaying plateau associated with the polarization relaxation process and background leakage. We can draw this conclusion because the U and D pulses contain only the flat plateau feature. This testing ensures that the base material is indeed ferroelectric with a distinguishable polarization switching current.

**Figure 2:**
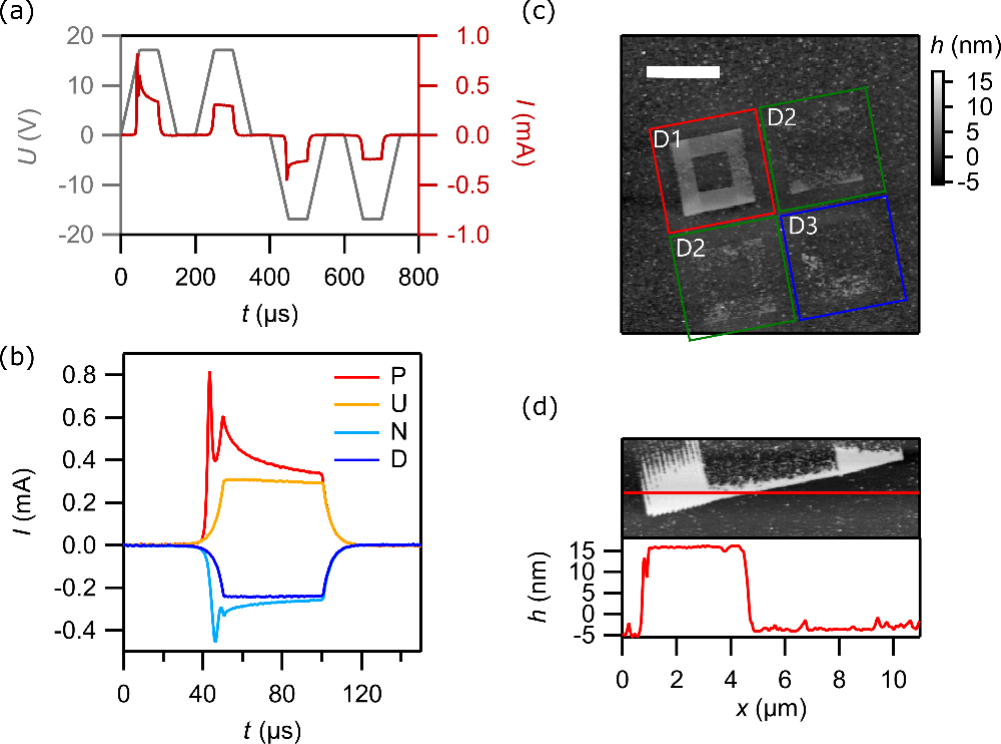
Characterization of AlBN ferroelectric and ULV-EBL patterned domains. (a) Positive-up and negative-down (PUND) measurements with applied voltage *U* and measured current *I* with respect to time. Four voltage pulses *U* with maximum and minimum +17 V and −17 V are applied across 20 nm AlBN through metal pads making contacts on top and below AlBN. The current *I* is measured in between the metal pads. (b) Current measured with 17 V PUND voltage pulses. The current differences in between P (or N) and U (or D) is the current that is coming from the polarization switching. (c) AFM height scan of four square rings with different electron-beam doses, after a KOH etching of 30 s. The red box is exposed with an area dose D1 = 51,200 μC/cm^2^. The green boxes are exposed with an area dose D2 = 25,600 μC/cm^2^. The blue box is exposed with an area dose D3 = 12,800 μC/cm^2^. The scale bar denotes 10 μm. (d) AFM topography after etching with KOH for 60 s. The upper is the AFM height image for the partial red region in (c). The lower is a line cut along the red line in the upper image for its height profile. The measured etch depth is 20 nm, which is the total AlBN thickness.

We next leverage the etch selectivity between the two different polarizations to confirm that the polarization is switched by ULV-EBL. With the N-polar AlBN sample where the polarization is pointing down (Figure 1(c)), the OH^–^ react with Al^+^ with eqs 1 and 2. However, in Al-polar configuration, due to the negatively charged dangling nitrogen bond (Figure 1(c)), which is supposed to be repulsive with OH^–^, the etching is prevented.^44,45^ This gives rise to the different etching speed reacting to the KOH:DI water liquid. We take advantage of this behavior to etch the sample with ULV-EBL electron-exposed and unexposed regions. To thoroughly electron-expose the 20 nm film, we use *V*_acc_ = 2 kV based on the Monte Carlo simulation (Figure 1e), which predicts electron penetration across the entire AlBN thickness. After that, an AZ400 K (active component KOH 1:4 with DI water) is used to etch the AlBN film with the expectation that ULV-EBL switched regions will be much more etch resistant than unswitched regions. The AFM scan shown in Figure 2(c) shows four features that were KOH etched for 30 s. The red box is exposed to an area dose of 51,200 μC/cm^2^. Green box ones are exposed with an area dose of 25,600 μC/cm^2^. The blue box is exposed to an area dose of 12,800 μC/cm^2^. After this etch treatment, only the highest dosed region retains the square box shape, suggesting switching. To further test this interpretation, the sample was KOH etched for another 30 s. Figure 2(d) shows a zoomed-in AFM topography image and a line scan across the highest electron-exposed feature (Figure 2(c,d)). The line scan shows a 20 nm step height, i.e., the entire film thickness, indicating the full etching of the AlBN (Figure 2(d)). This is consistent with the other reports that the C– surface has a much faster etching speed than the C+ surface of ferroelectric materials,^38,44,45^ confirming local and patternable electron beam induced switching.

We then explore the writing resolution and how the dose affects the measured width of line features by exposing a series of lines at varying dose and characterizing them with AFM. Figure 3 shows two different fine features: one-dimensional lines and a lattice made by dots. The lines in Figure 3(a) have different doses. Line D_*n*_ has a dose of *D*_*n*_ = 2^*n*–1^ × 100 pC/cm. Higher doses led to wider exposure (Figure 3(b)). This is understandable with the lateral distributions of electrons in interaction with the material. The smallest line width *w* is 35 nm. Resolution is limited by both the e-beam resolution and possibly the smallest ferroelectric grain size. Figure 3(c) shows the PFM phase image of a square lattice with doses ranging from 0.01 (top left) to 4 pC (bottom right).

**Figure 3:**
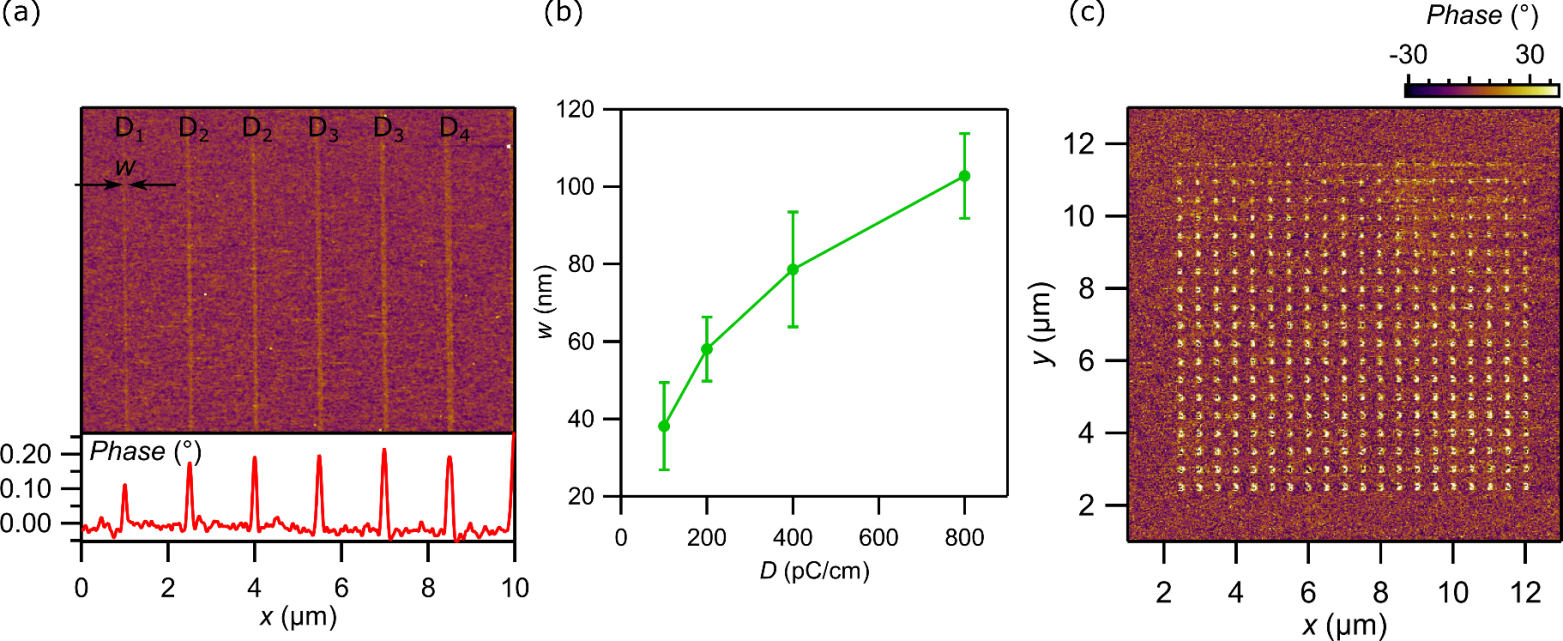
ULV-EBL ferroelectric switching resolution. (a) AFM phase image of a series of lines patterned with different doses. Lines D_1_, D_2_, D_3_, and D_4_ are exposed with doses of 100, 200, 400, and 800 pC/cm, respectively. The lower graph is a line cut of the upper image. (b) Line width *w* collected from lines in (a) with respect to their exposure dose *D*. (c) PFM phase image of a square lattice with dose gradient ranging linearly from 0.01 pC (top left) to 4 pC (bottom right) with 0.01 pC per step per dot.

To integrate the AlBN ferroelectric with vdW materials, a monolayer graphene/hBN device (Figure 4(a,b)) is fabricated onto the 20 nm AlBN substrate (see Methods). The hBN thickness is ∼10 nm for sample homogeneity. A 49 nm thick tungsten (W) layer underneath the AlBN serves as the gate of the ferroelectric field-effect transistor. For this device, an acceleration voltage *V*_acc_ = 5 kV is chosen, based on Monte Carlo simulations, so that the electron beam penetrates through the vdW stack and switches the polarization of the ferroelectric thin film. The electron beam current is *I*_b_ = 0.46 nA and the exposure dose is *D* = 50,000 μC/cm^2^. The graphene layer is kept grounded during the exposure. A solid region is exposed by ULV-EBL (dashed line in Figure 4(a)). It is labeled the “exposed” region (e) in comparison with the “unexposed” region (u), where there is no ULV-EBL exposed on that half Hall bar region. *In situ* graphene transport is performed, while the device is monitored in the ULV-EBL chamber. By applying the gate voltage *V*_g_ within the coercive field range, we measured graphene resistance *R* to identify the charge neutrality. Lead 6 is connected to lead 2; thus, in the exposed side we process a three-terminal measurement (see Supporting Information for details).

**Figure 4:**
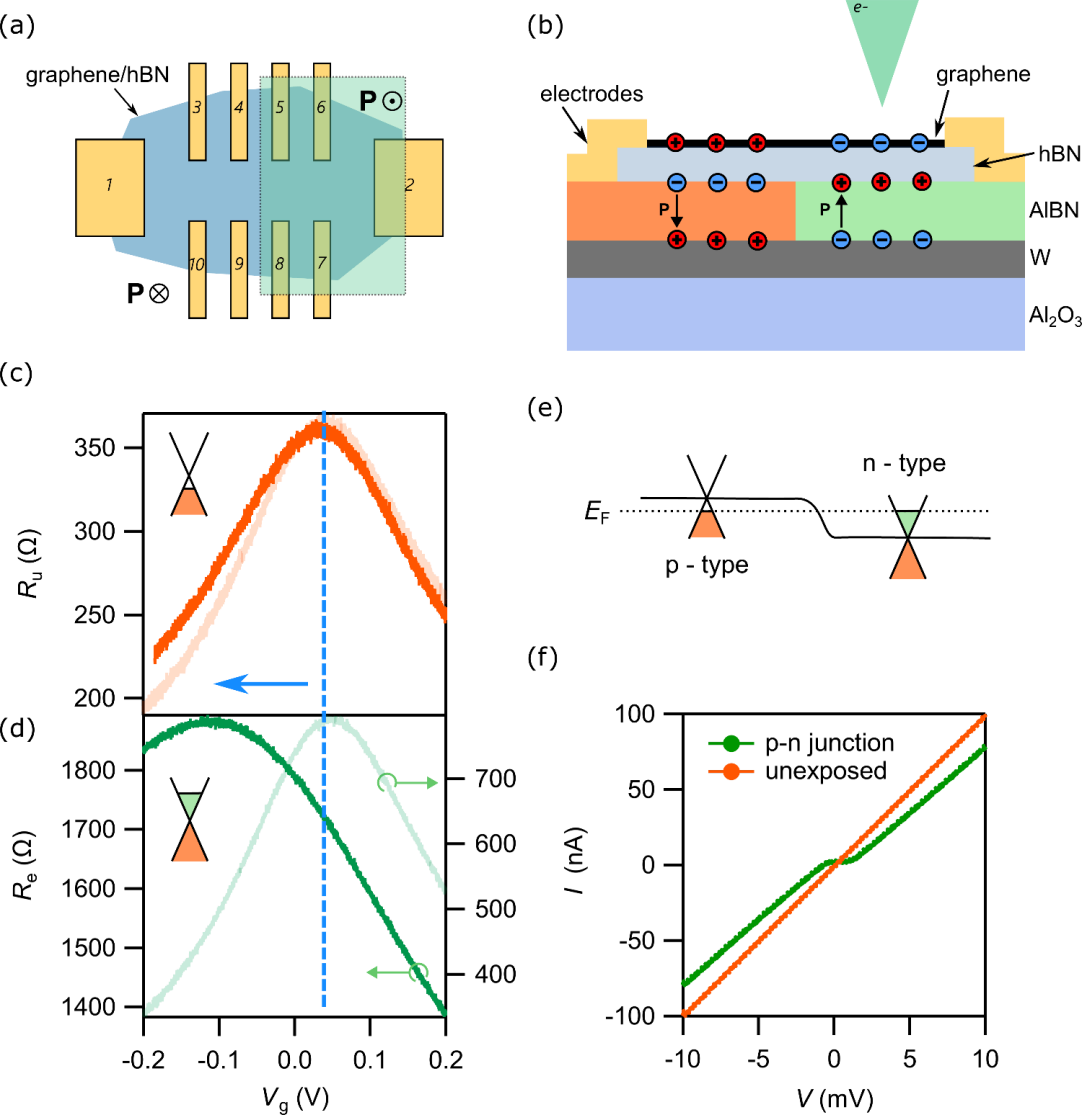
ULV-EBL ferroelectric switching on graphene. (a) Schematic diagram of the device. The blue region denotes the graphene/hBN device. There are electrical contacts to the graphene (yellow rectangles). The region enclosed by a dashed line is the ULV-EBL exposure region (transparent green color). The rest of the Hall bar is the no exposure region. (b) Schematic diagram of the device geometry. A monolayer graphene and 10 nm thick hBN vdW stack is placed on top of an AlBN/W/sapphire substrate. The AlBN is as-grown polarization-down state (orange region). The electron beam exposed region has polarization pointing up (green region). Different polarization induces different types of doping carriers in graphene, shown as electrons (blue circle) and holes (red circle). (c, d) *R*–*V*_g_ measurement before (light color line) and after (opaque line) the exposure to show the Dirac point for the unexposed (*R*_u_) and exposed (*R*_e_) region. The blue dotted line denotes the original Dirac point position, and the blue arrow shows the direction of the shift. *T* = 300 K. (e) Illustration of the p–n junction energy band. Dashed line shows the Fermi energy, *E*_F_. (f) *I*–*V* curves for the p–n junction and the unexposed region as a comparison. *T* = 15 mK.

Prior to exposure, graphene charge neutrality in *R*_e_ ≡ *V*_5–6_/*I*_1–2_ and *R*_u_ ≡ *V*_3–4_/*I*_1–2_ is observed at *V*_g_^CNP^ = +0.08 V for both, showing that graphene is initially hole doped (Figure 4(c)). This observation is consistent with the fact that the ferroelectric thin film underneath the graphene is prepolarized with a downward pointing polarization, implying the presence of negative surface charge that dopes graphene to p-type. Upon exposure, the exposed regions *R*_e_ exhibit the charge neutrality at *V*_g_^CNP^ = −0.1 V, while the charge neutrality position in the unexposed areas remains unchanged. The difference in *V*_g_^CNP^ in the exposed region is attributed to the alteration of the polarity in the regions irradiated by the electron beam, transitioning from a downward to an upward polarization. The resulting positive surface charge subsequently dopes graphene into an n-type (Figure 4(c,d)). By comparing the mobility before and after the exposure, we confirm that the method does not deteriorate to graphene quality (see Supporting Information 8). Combined with the magnetotransport results, the corresponding change in carrier density upon ULV-EBL exposure is about 1.77 × 10^11^ cm^–2^ (see Supporting Information 2).

The patterning shown from Figure 4(c) and (d), where the device is half exposed and half unexposed, results in a p–n junction behavior in graphene when *V*_g_ is applied such that the exposed region is n-doped and the unexposed region is p-doped. Figure 4(e) shows a schematic diagram of the p–n junction energy band in graphene. Using *I*–*V* curve characterization, the p–n junction displays a diode-like barrier, in contrast to the linear current to voltage relation observed for the unexposed region (Figure 4(f)). Four-terminal *I–V* measurements confirm that the nonlinearity is not from the contact such as the Schottky barrier (see Supporting Information 5 and Figure S6). Massless Dirac Fermions in graphene are predicted to exhibit perfect transmission through potential barriers at normal incidence due to Klein tunneling. Due to the oblique scattering angles across the p–n junction and the nonideal Hall bar geometry of the device, we do not observe reflectionless tunneling in our device. The effective barrier observed here is consistent with the constraint of momentum conservation across the junction (see discussions in Supporting Information 5).^46−49^

Manipulating the electrical and optical properties of vdW materials is important for expanding the range of properties and exploring new physics within these materials. Moiré engineering represents a powerful method for creating superlattices^3,5,50−52^ and has shown great success in uncovering various novel condensed matter phases. This method is limited to generating periodic potentials, which restricts the variety of structures that one can explore. The method described here offers top-down programming and allows the ability to create a wider range of both periodic and aperiodic structures with the ability to combine them in a single device.

The specific top-down approach developed here, involving a buried ferroelectric layer that is programmed using ULV-EBL to achieve electrostatic patterning of a vdW layer, offers many advantages. The programming step involves no cleanroom processing steps or e-beam resist and is indefinitely stable after programming, unlike previous approaches involving LaAlO_3_/SrTiO_3_, where the programming is metastable.^1,53^ “Programmable” here means one can create any desired polarization pattern from a given initially polarized state. Here, the ferroelectric field effect is demonstrated in graphene, but it should be applicable to most 2D materials, e.g., TMDs. Here we do not demonstrate reconfigurability, e.g., erasure and rewriting of ferroelectric patterns. As the e-beam exposure can only switch AlBN polarization from the down to up orientation, reconfigurability can be achieved by adopting the back-electrode to define the global polarization electrically.

The high spatial resolution afforded by ULV-EBL should enable periodic superlattices whose geometries are bounded only by the lithographic limit, thus providing new access to engineered bands and designer electronic phases. A future target is creating a platform for 2D analog quantum simulation and thus predicting phases that have been discovered using other methods like moiré interference. Unlike the twistronic approach, diverse device properties can be mixed and matched from a single base material.

The experimentally measured carrier concentration in our devices is orders of magnitude lower than the surface charge measured from bulk measurements.^31^ To account for this discrepancy, we consider several factors. Surface physisorption from charged species can reduce the carrier concentration. The dielectric permittivity of the bottom hBN layer can also screen the ferroelectric polarization. It is this same screening effect that motivates researchers to use hBN in the first place, in that it screens inhomogeneities and improves the overall quality of the graphene by providing an atomically flat, clean, and chemically inert interface.^54−56^ To balance the trade-off between the benefits of hBN and its screening effect on the ferroelectric field, we attempted to minimize the thickness of this hBN spacer. The use of fully hBN-encapsulated devices with an ultrathin bottom hBN could ideally increase the induced carrier concentration and prevent the charge compensation from other sources. Similar passivation mechanisms may be present to reduce the net surface charge,^57^ such as in LiNbO_3_, where charge compensation via surface reconstruction may be favored.^58^ In addition, the charge trapping effect^59,60^ is likely to manifest in graphene owing to the elevated surface charge density in AlBN and the thin hBN thickness.

In conclusion, we demonstrate the use of ULV-EBL to pattern the ferroelectric Al_1–*x*_B_*x*_N thin film, where the electron beam energy and dose are optimized to generate domains as small as 35 nm, with a lower limit yet to be determined. The electron penetration depth is controlled precisely by *V*_acc_, allowing one to regulate the electron dose in all three directions to minimize exposure of fragile substrates or other integrated layers that may experience deleterious effects. The electron-beam-written polar pattern, and its compensating surface charge pattern, can locally tune the charge density within an adjacent vdW layer with resolution on the order of 10s of nanometers. The method can be generalized and can apply to other 2D materials with different programmable substrates. These observations and capabilities demonstrate the first and enabling step toward a new platform for a solid-state-based 2D analog quantum simulator.

## Methods

### ULV-EBL

In the experimental procedure, ultra-low-voltage electron beam lithography (ULV-EBL) was conducted using a Zeiss Gemini SEM 450 scanning electron microscope (SEM), equipped with a high-speed 20 MHz Raith Elphy Plus lithography pattern generator. The electron beam acceleration voltage ranged from 100 V to 30 kV. Unintended exposure was carefully avoided by making the adjustment and alignment of focus and stigmation on a standard Chessy chip. The ULV-EBL three-point alignment (TPA) and write-field alignment (WFA) were carried out at the specially designed markers by using photolithography at the edge of the sample far away from the Hall bar region. Then, the beam was blanked and shuttled to the Hall bar to expose the designed pattern. The exposure was in vacuum with the chamber pressure 8.5 × 10^–7^ mbar. The exposure working distance was 2.5 to 4 mm with the beam current varying from 57 to 460 pA. The dwell point step size was set to 10 nm.

### AFM and PFM

AFM and PFM imaging were carried out using a commercial AFM system (Asylum Research MFP-3D). AFM tips for AFM AC and contact scans was made of doped silicon (Aspire Conical AFM Tips CFMR-25) with an in-air resonance frequency of 75 kHz and 3 N/m spring constant. PFM was done with a PtIr_5_-coated AFM probe from Nanosensors (PPP-EFM-50) with an in-air resonance of 75 kHz. The PFM image was done by driving the tip at a frequency in the range from 340 to 370 kHz while engaging the sample surface and a driving voltage of 2 to 5 V. Our AFM and PFM imaging was performed under ambient conditions in a humidity- and temperature-stabilized environment (50% RH).

### Device Fabrication

*AlBN/W/Al*_2_O_3_*Growth*: The commercial (001) Al_2_O_3_ substrate (Jiaozuo TreTrt Materials) was cleaned in isopropyl alcohol and methanol. Then a layer of 40 nm W (110) was grown on top while holding the substrate temperature at 300 °C using magnetron sputter DC sputtering under Ar conditions. An AlBN film was deposited in the same chamber with reactive pulsed DC sputtering from an Al target and rf sputtering from a B target.^30−32^

*Graphene/hBN Device*: The graphene/hBN heterostructure was stacked by a modified dry transfer process based on polycaprolactone (PCL),^61^ with a drop-down temperature at 80 °C. First, a monolayer graphene flake was exfoliated and identified on a SiO_2_/Si substrate under an optical microscope and picked up by a PCL stamp, followed by a thin hBN flake (thickness ∼10 nm) that served as an intermediate layer between the graphene and the AlBN substrate to improve sample homogeneity. Standard e-beam lithography was then used to define contacts to graphene; Cr/Au (2/100 nm) electrodes were deposited by e-beam evaporation to connect graphene and conductive pads to enable bonding to the sample. We employed silver-epoxy-based bonding to prevent punching through the 20 nm AlBN layer.

### KOH Etching

We use photoresist developer AZ400K, whose active component is KOH 1:4 with water to etch the AlBN, simply by an AZ400K bath at room temperature. Under an alkaline condition, for example KOH:DI water liquid here, there are reactions happening as
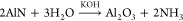
1
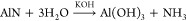
2where KOH is the catalyst shifting the equilibrium to the right.

### Electrical Measurements

At room temperature, there is customized electrical feedthrough in ULV-EBL allowing *in situ* electrical measurement in the SEM chamber. The measurement channels consist of NI DAQ hardware (PXI-4461) and Krohn-Hite preamplifiers (model 7008). The Dirac point shift measurement is a lock-in measurement (frequency = 13 Hz) at room temperature. The sample was then transferred into a dilution refrigerator (Leiden MNK) and cooled to 15 mK. A DC measurement for *I*–*V* characterization of the p–n junction effect was carried out at the base temperature.

## Supporting Information Available

Additional experimental details, characterizations, methods, and supplementary figures, including Monte Carlo simulation, Landau fan measurements of graphene, additional PFM images, AlBN surface roughness, p–n junction characterizations, XRD characterization of AlBN, PUND and PE measurement, graphene mobility before and after the exposure (PDF)
