# Supplementary material for: Nanoscale Ferroelectric Programming of van der Waals Heterostructures
Source: Nano Lett. 2024 Dec 13;24(51):16231–8. doi: 10.1021/acs.nanolett.4c03574 (PMC11673570; doi:10.1021/acs.nanolett.4c03574)
Supplement: Supplementary file 1 — nl4c03574_si_001.pdf [file nl4c03574_si_001.pdf]

Nanoscale ferroelectric programming of van der Waals heterostructures

Supporting Information

Dengyu Yang[1,2,3], Qingrui Cao[1,2], Erin Akyuz[1,2], John Hayden[4], Josh Nordlander[4], Ian Mercer[4], Muqing Yu[2,3], Ranjani Ramachandran[2,3], Patrick Irvin[2,3], Jon-Paul Maria[4], Benjamin Hunt[1,2,], and Jeremy Levy[2,3]

[1] Department of Physics, Carnegie Mellon University, Pittsburgh, Pennsylvania 15213, USA
[2] Pittsburgh Quantum Institute, Pittsburgh, Pennsylvania 15260, USA
[3] Department of Physics and Astronomy, University of Pittsburgh, Pittsburgh, Pennsylvania 15260, USA
[4] Department of Materials Science and Engineering, The Pennsylvania State University, Pennsylvania 16802, USA

Contents

1 Monte Carlo Simulation **2**

2 Landau fan measurements of graphene **3**
2.1 Unexposed region 3
2.2 Exposed region 3

3 More PFM scan images **3**

4 AlBN surface roughness **4**

5 p-n junction characterizations **5**

6 XRD characterization of AlBN **7**

7 PUND and PE memasurement **7**

8 Mobility before and after the exposure **7**

1 Monte Carlo Simulation

We use CASINO Monte Carlo simulation [1, 2, 3] to simulate the trajectories (Fig. S1 (a, b)) and determine the optimal electron acceleration voltage used V_{acc} to energize the electrons. To penetrate most of the thickness of the AlBN film, a $V_{\mathrm{acc}} = 500$ V is used to expose the 11 nm AlBN film (Fig. S1 (a)). And a $V_{\mathrm{acc}} = 1$ kV is used to expose the 20 nm AlBN film (Fig. S1 (b)). In Figures S1 (a, b), the green lines show the trajectories of the penetrated electrons and the red lines show the backscattered electrons. Brighter colors denote higher energy and darker colors mean lower electron energy. In both cases, the electron energy is sufficient enough to switch the surface polarization and be seen under AFM. Figures S1 (c, d) show the normalized stop hits of the electrons with respect to depth Z. For the 11 nm AlBN film ($V_{\mathrm{acc}} = 500$ V) case, the electrons mostly stop before the AlBN/W interface. As in the case of 20 nm AlBN film ($V_{\mathrm{acc}} = 1$ kV), the electrons reach W but the electron energy decreases significantly due to the higher density of the material and the larger atomic number.

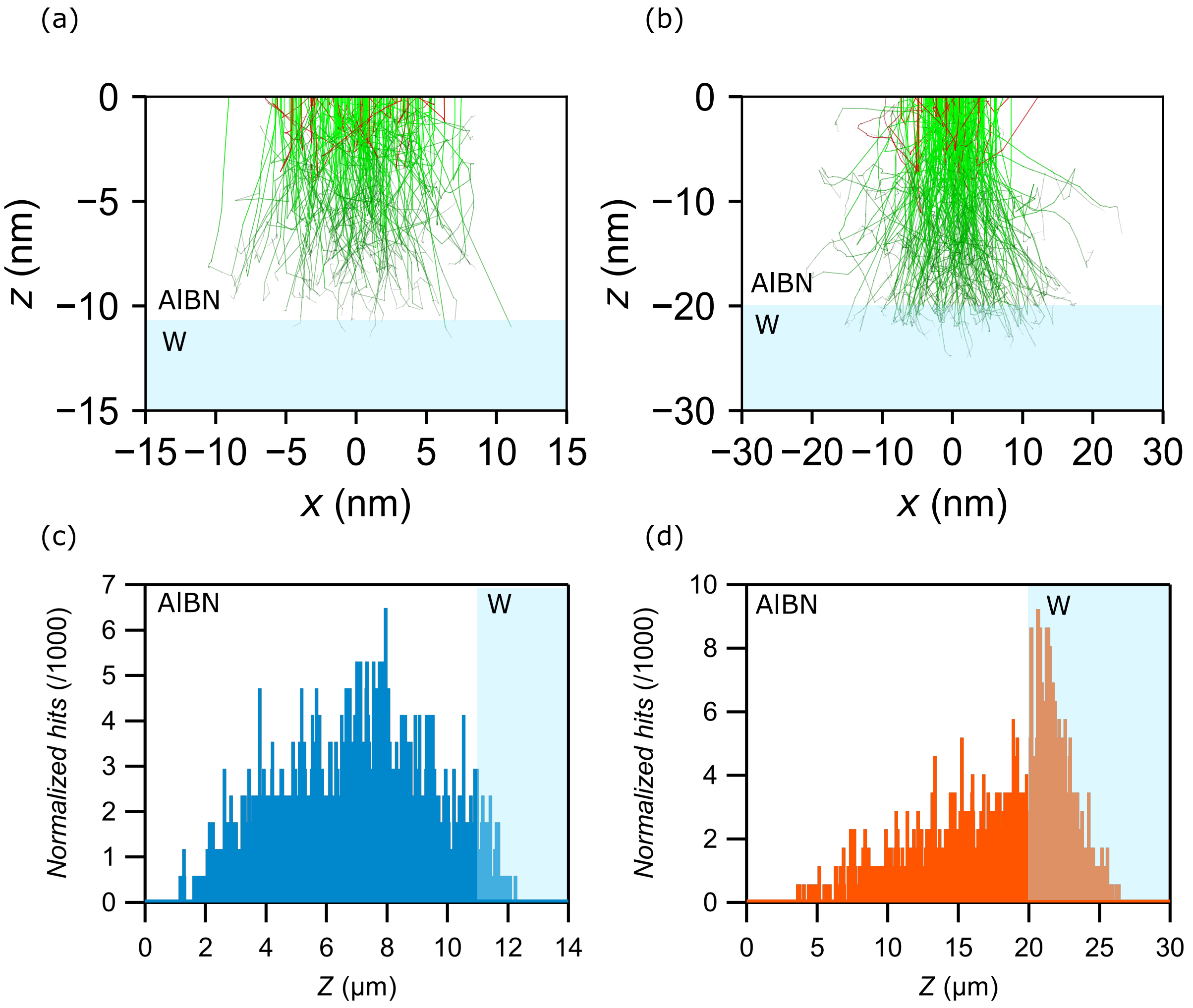

Fig. S1: **Monte Carlo simulation of ULV-EBL ferroelectric switching penetration depth** (a) Monte Carlo simulation for electron trajectories of a 500 V acceleration voltage in an 11 nm AlBN film. (b) Monte Carlo simulation for electron trajectories of a 1 kV acceleration voltage in a 20 nm AlBN film. (c) Normalized electron stop-hit distribution with respect to Z for a 500 V acceleration voltage at an 11 nm AlBN film. (d) Normalized electron stop-hit distribution with respect to depth Z for a 1 kV acceleration voltage at a 20 nm AlBN film. The light blue regions denote the W layer underneath the AlBN.

2 Landau fan measurements of graphene

As a standard method to characterize the properties of the graphene, transport measurements of longitudinal resistance R_{xx} and Hall resistance R_{xy} in the quantum Hall regime are carried on the device and the Landau fan diagram is measured by applying the perpendicular magnetic field B up to ± 18 T. The experiment is carried out at a dilution refrigerator, Leiden MNK, with a base temperature < 15 mK. Due to the imperfect Hall geometry of the device, we measure the Landau fan diagram of both positive and negative magnetic field up to 18 T, and then carry out a symmetric analysis to both longitudinal resistance R_{xx} and Hall resistance R_{xy}, where $R_{\mathrm{xx}}(B) = 1/2(R_{\mathrm{xx}}(B) + R_{\mathrm{xx}}(-B))$ and $R_{\mathrm{xy}}(B) = 1/2(R_{\mathrm{xy}}(B) - R_{\mathrm{xy}}(-B))$.

2.1 Unexposed region

Figure S2 shows the graphene Landau fan diagram of the unexposed region on the AlBN substrate. The longitudinal resistance $R_{\mathrm{xx}} = V_{\text{3-4}}/I_{\text{1-2}}$ and the Hall resistance $R_{\mathrm{xy}} = V_{\text{3-10}}/I_{\text{1-2}}$ are measured. Landau levels of $\nu = \pm 1, \pm 2$ can be observed. The Dirac point is observed at $V_{\mathrm{g}}^{\mathrm{CNP}} = +0.12$ V. Compared with the data shown in the main text where the Dirac point is taken at 300 K, it shifts to the electron doping region a little. This is due to the device geometry which is an open-faced graphene device. When transferring from the ULV-EBL to the DR fridge, we need to break the SEM vacuum, transfer it in air and then load it into the DR fridge vacuum. The process that exposure to the air, introduces contact with the charges in the atmosphere so which shifts the Dirac point.

At $V_{\mathrm{g}} = -0.2$ V (0.32 V away from the charge neutrality $V_{\mathrm{g}}^{\mathrm{CNP}}$), we observe the $\nu = \pm 2$ Landau gap at $B = \pm 6.5$ T, corresponding to a carrier density of $|n| = \frac{|\nu e B|}{h} \approx 3.14 \times 10^{11}\ \mathrm{cm}^{-2}$. Based on this estimation, we deduce that the shift upon ULV-EBL writing ($|\Delta V_{\mathrm{g}}| = 0.18$ V, see Fig. 4 in main text) is about $1.77 \times 10^{11}\ \mathrm{cm}^{-2}$. Since the thicknesses of the $\mathrm{Al_{1-x}B_xN}$ substrate and hBN are 20 nm and 12 nm, respectively, with the hBN dielectric constant $\epsilon_{\mathrm{hBN}} = 4$, we can further estimate that $\epsilon_{\mathrm{AlBN}} \approx 7.6$ using two parallel plate capacitors in series.

2.2 Exposed region

In the exposed region, $R_{\mathrm{xx}} = V_{\text{5-6}}/I_{\text{1-2}}$ and $R_{\mathrm{xy}} = V_{\text{5-8}}/I_{\text{1-2}}$ (Fig. S3). Lead #7 is not working and lead #6 and lead #2 are connecting during fabrication process. It turns out the $R_{\mathrm{xx}} = V_{\text{5-6}}/I_{\text{1-2}}$ here is a three-terminal measurement. Because of the switched ferroelectric polarization, Dirac point is now at $V_{\mathrm{g}} =$ -50 mV showing in R_{xx} at zero field (Fig. S3 (c) bottom inset). But in R_{xy} results, we cannot see a clear Dirac point position accordingly. That is because the R_{xy} measured from lead #5 and #8 are too close to the separation edge between the exposed and unexposed region. Due to the imperfect Hall geometry of the device, it is hard to separate the exposed and unexposed results from R_{xy}. There is also a hint to this where the R_{xy} Landau fan shows some different slopes that can be traced to a position different from the charge neutrality point (CNP)(Fig. S3 (d)) indicating that the measured result is a mixing of two carrier density regions.

3 More PFM scan images

We use piezoelectric force microscopy (PFM) to characterize the ULV-EBL patterned ferroelectric domains. As Figure S4 shows, a square annulus and the letter “P” are exposed using ULV-EBL onto 20 nm thick AlBN thin film on top of W and sapphire substrate. Under PFM amplitude (Fig. S4 (a, c)) and phase (Fig. S4 (b, d)) images, the exposed annulus can be seen clearly. The amplitude gives a height change of near 30 pm coming from the piezoelectric response. The ULV-EBL switched ferroelectric domain shows a larger than 50 degree phase difference comparing with the AlBN intrinsic polarization inheriting from sample growth.

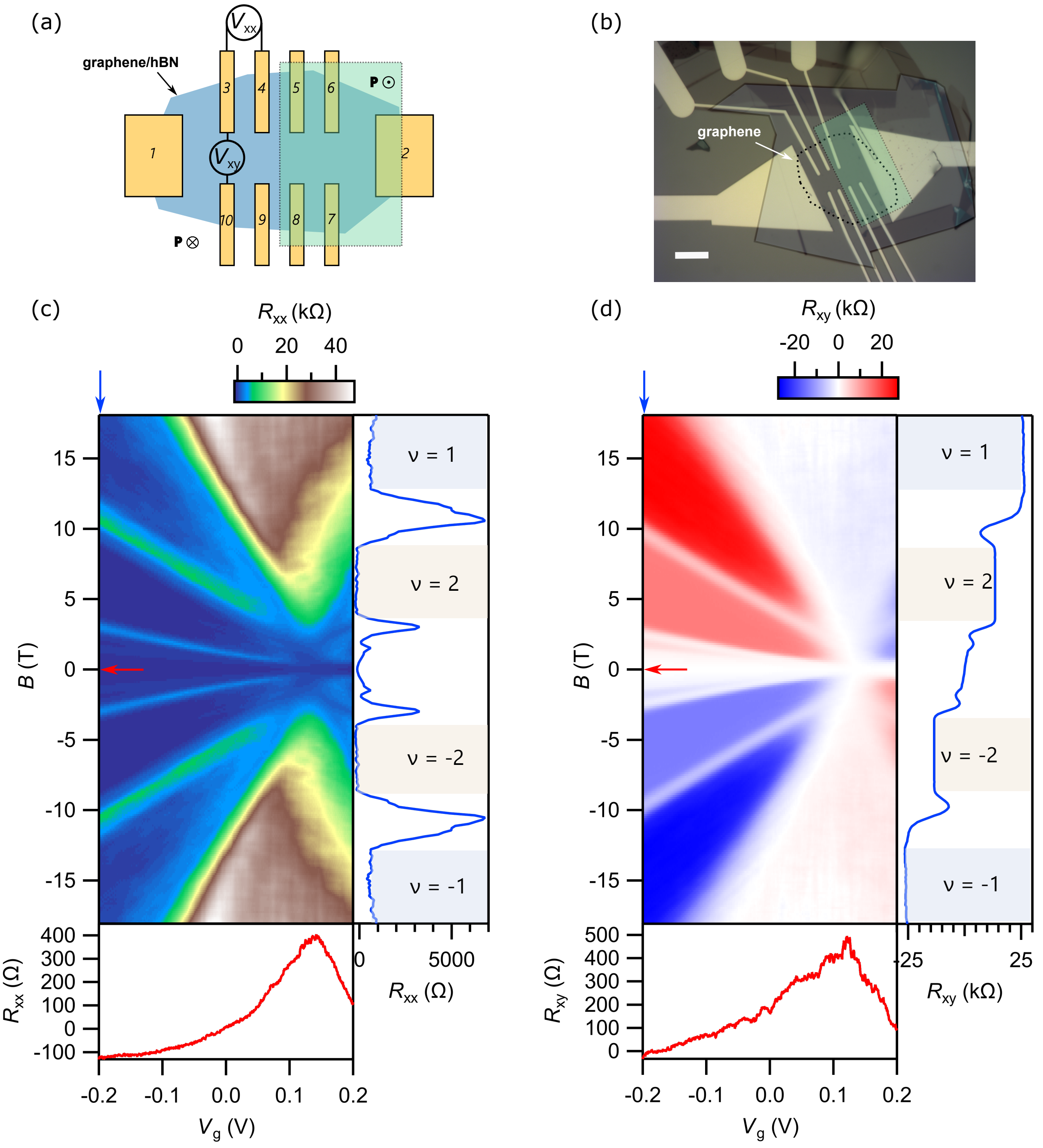

Fig. S2: **Graphene Landau fan measurement for unexposed region** (a) Schematic diagram of the device. The blue region denotes the graphene/hBN device. There are electrical contacts to the graphene. The region covered in the dashed line is the ULV-EBL exposure region. The rest of the Hall bar is the unexposed region. The voltages of R_{xx} and R_{xy} are measured in electrode pairs of V_{xx} and V_{xy}.

(b) Optical image of the device. Solid black line denotes the region of top hBN. The region covered in the dashed line under green color is the ULV-EBL exposure region. The scale bar denotes 10 μm. (c) Longitudinal resistance R_{xx} as a function of the magnetic field B and the gate voltage V_g measured below 15 mK. (d) Hall resistance R_{xy} as a function of the magnetic field B and the gate voltage V_g measured below 15 mK. In (c) and (d), right insets are a linecut taking at the blue arrow position (V_g = -0.2 V) along magnetic field B, bottom insets are a linecut taking at the red arrow position ($B = 0$) along the gate voltage V_g.

4 AlBN surface roughness

We perform atomic force microscopy (AFM) to analyze the surface morphology and roughness. The AFM topography image (Fig. S5) reveals a smooth surface with a root-mean-square (RMS) roughness of approximately 290 pm over a 30 µm × 30 µm area.

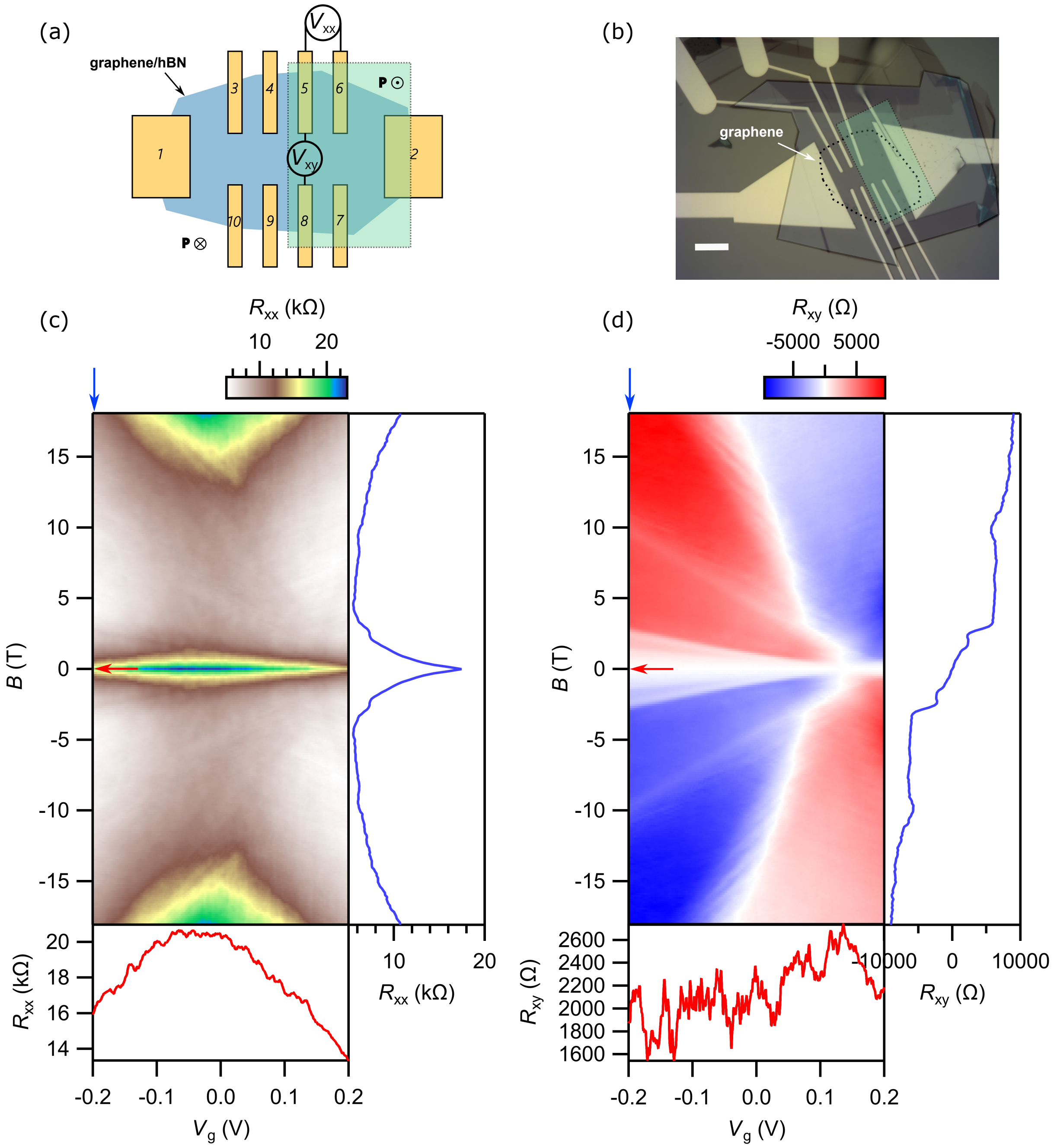

Fig. S3: **Graphene Landau fan measurement for exposed region** (a) Schematic diagram of the device. The blue region denotes the graphene/hBN device. There are electrical contacts to the graphene. The region covered in the dashed line is the ULV-EBL exposure region. The rest of the Hall bar is the unexposed region. The voltages of R_{xx} and R_{xy} are measured in electrode pairs of V_{xx} and V_{xy}. (b) Optical image of the device. Solid black line denotes the region of top hBN. The region covered in the dashed line under green color is the ULV-EBL exposure region. The scale bar denotes 10 μm. (c) Longitudinal resistance R_{xx} as a function of the magnetic field B and the gate voltage V_g measured below 15 mK. (d) Hall resistance R_{xy} as a function of the magnetic field B and the gate voltage V_g measured below 15 mK. In (c) and (d), right insets are a linecut taking at the blue arrow position (V_g = -0.2 V) along magnetic field B, bottom insets are a linecut taking at the red arrow position ($B = 0$) along the gate voltage V_g.

5 p-n junction characterizations

We observe the nonlinear IV curve from across the p-n junction patterned with the ferroelectric programming method. To check if the non-linearity is coming from lead or contact for example Schottky barrier, we perform the four-terminal IV measurement which eliminate the effect from leads. From the four-terminal IV, the non-

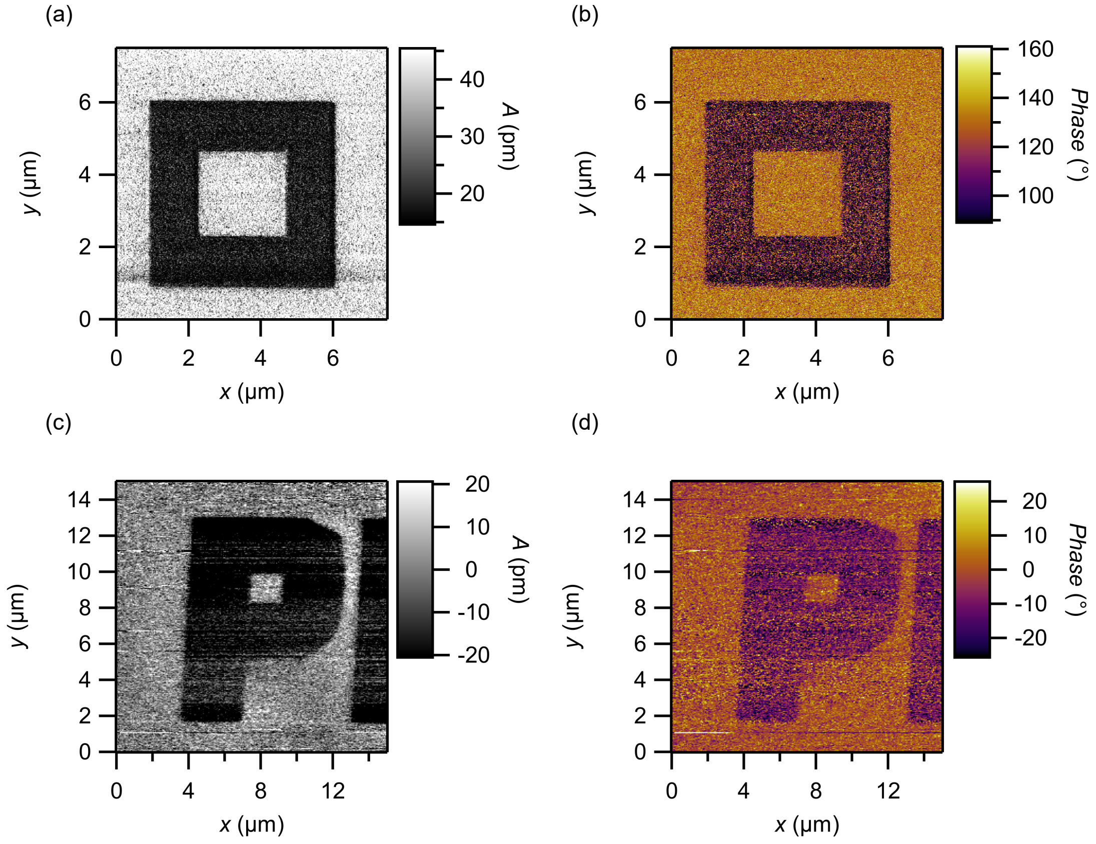

Fig. S4: **PFM images** (a) PFM amplitude image. (b) PFM phase image. (c) PFM amplitude image. (d) PFM phase image.

linearity is still observed, this means it is a effect from the graphene device, not the leads.

To better understand the current-voltage (IV) characteristics, we aim to model the p-n junction in graphene. A common approach involves considering the transmission coefficient [4, 5, 6, 7], often simplifying calculations by assuming a high barrier limit where the barrier height significantly exceeds the electron energy. However, in our case, the shift in the Dirac point is relatively small—approximately 1.77×10^{11} cm^{-2}—resulting in an energy difference between the p-type and n-type regions of about 67 meV. This energy barrier is comparable to the source voltage used in our IV measurements, making the high barrier approximation unsuitable here.

The resistance measured in graphene consists of two components: ballistic and diffusive contributions. The difference in resistance (R_{odd}) between scenarios with a p-n junction (R_{p-n}) and without one (R_{p-p}) arises entirely from scattering at the junction. By comparing the resistance before and after exposure, we estimate R_{odd} to be around 1000 Ω in our case. Incorporating this value into the theoretical model suggests the presence of a characteristic width d for the p-n junction, analogous to the depletion region in conventional semiconductor p-n junctions. This width is on the order of hundreds of nanometers to a few micrometers. Such a relatively large d complicates the determination of whether ballistic or diffusive transport dominates the resistance. This is because the diffusive carrier density $n_{diff} = e/h\mu$ is similar to the the gradient of carrier concentration $n' = n/d$, making the coefficient β between them close to 1. Under these conditions, the approximate transition probability $T = cos^2(\theta)$ here with an incident angle $\theta = 1°$ yielding 99.9%, does not align with our observations.

We then consider the momentum change of carrier across the junction. The momentum component $k_y = k_F \sin\theta$ along the junction is conserved, while the normal component k_x is given by $k_x = \sqrt{(E(V_s)/\hbar v_F)^2 - k_y^2}$, where $E(V_s)$ is the Fermi energy of charge carriers determined by the source voltage V_s [4, 5, 6, 7]. Due to the 1.77×10^{11} cm^{-2} carrier concentration shift in between the p and n type doping region, the energy barrier for the p-n junction is ~67 meV. In real devices, the junction is not a sharp-step-function-like energy barrier, but a gradual change within a certain distance d as established before. Previous studies have shown that, even though there is no band gap in graphene, a band gap/barrier like feature emerges when k_x becomes imaginary.

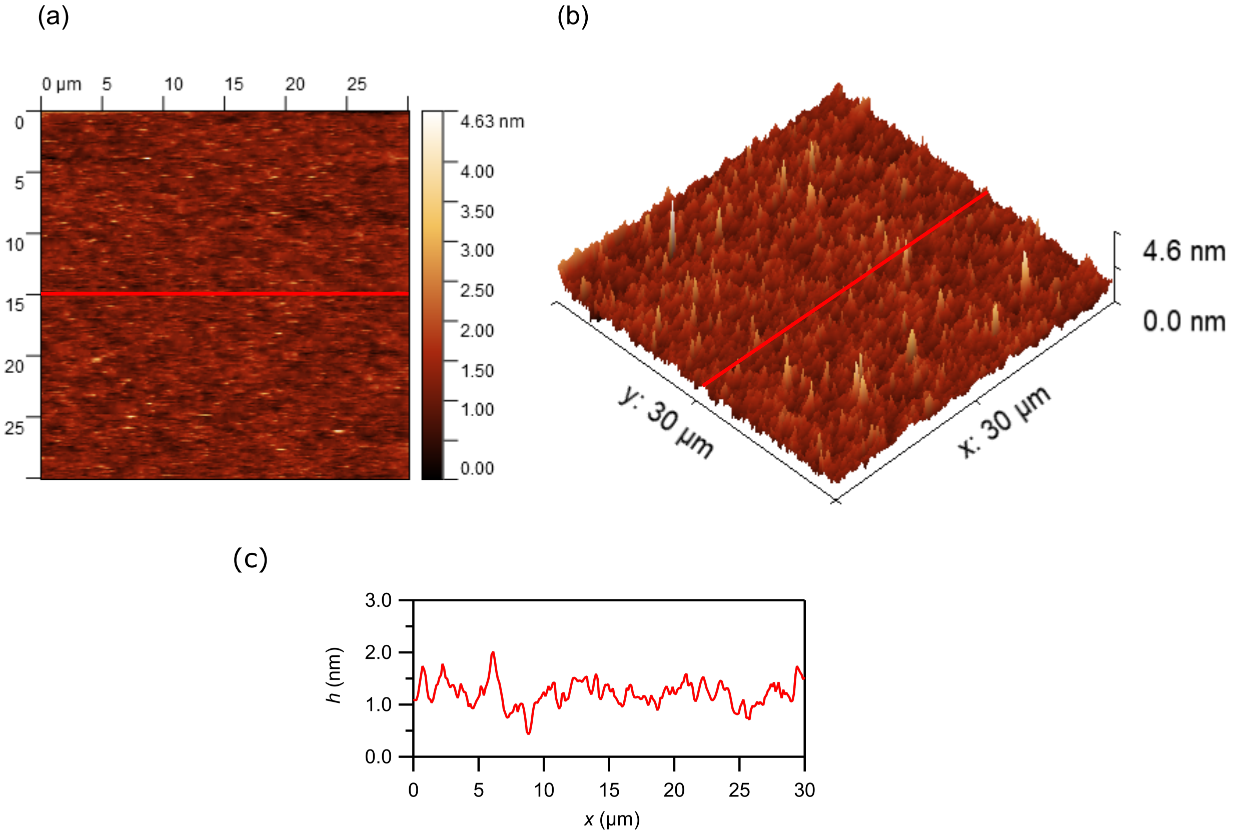

Fig. S5: **AFM images on surface morphology** (a) AFM contact mode scan height image. (b) 3D view of AFM contact mode scan height image. (c) Linecut at the red line in (a) and (b).

Therefore, we can observe a band gap by determining the source voltage V_s that renders k_x imaginary. This condition occurs when $|V_s| < \frac{\hbar v_F k_F \sin\theta}{e} \approx 0.8\text{mV}$, where the Fermi velocity $v_F \approx 1 \times 10^6\text{m/s}$ and the Fermi wavevector $k_F = \sqrt{n\pi} \approx 5.6 \times 10^8\text{m}^{-1}$. This calculation matches the order of magnitude of the barrier we observe experimentally. The discrepancy between the calculated and measured values may result from imperfections in the device geometry and from measurement inaccuracies due to the small size of the barrier.

6 XRD characterization of AlBN

To better understand the AlBN structure, we perform the X-ray diffraction (XRD) to the 20nm AlBN/W on sapphire substrate. As Figure S7 shows, there is a dominant AlBN (002) peak, indicating the c-axis orientation. The XRD shows it has polycrystalline texture.

7 PUND and PE memasurement

To measure the polarization on this thin AlBN films, we perform the PUND measurement with triangle voltage pulses. From the PUND measurement, we observe up to 150uC/cm^2 polarization as shown in Figure S8.

8 Mobility before and after the exposure

To estimate the mobility before and after the exposure, we need to look at the four-terminal longitudinal resistance of the device before and after the exposure. Due to the reality of the device, there is no longitudinal voltage pair can be found in the exposed region. Figure S9 shows the change of four-terminal resistance with respect to the back-gate voltages at 300K, the four-terminal voltage is measured in the same pair of leads 3 and 5 which across the junction (Fig. S6 (a)). The source and drain is from lead 1 to 2 (Fig. S6 (a)). We then plot the conductivity (σ) vs carrier concentration (n) as Figure S9 (b) shown. To get the mobility, we apply the

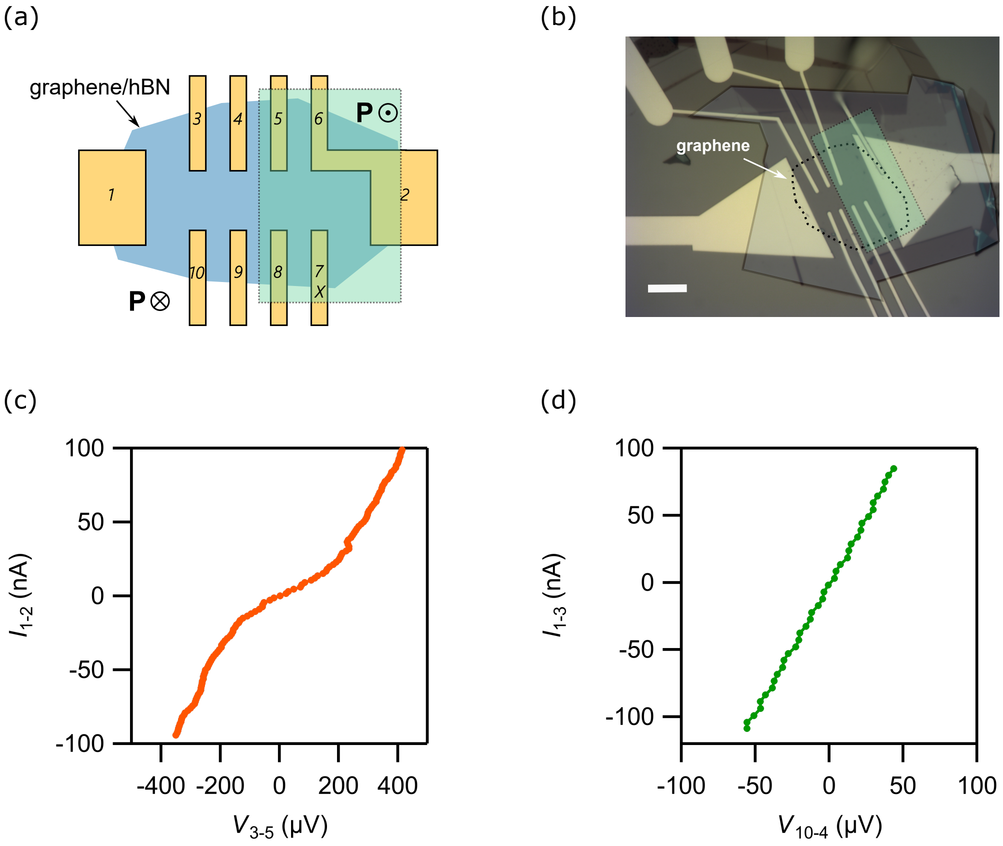

Fig. S6: **Four-terminal IV characterization.** (a) Schematic diagram of the device. (b) Optical image of the device. The scale bar denotes 10 μm. (c) 4-terminal IV curve across the junction. (d) 4-terminal IV curve in the unexposed region.

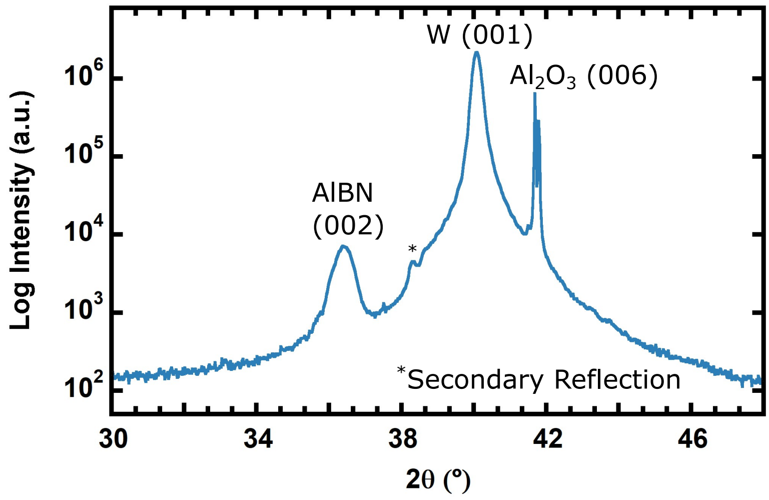

Fig. S7: **X-ray diffraction**

Drude formula $\sigma = \mu en$ and calculate the slope of the conductivity (σ) vs carrier concentration (n). From our

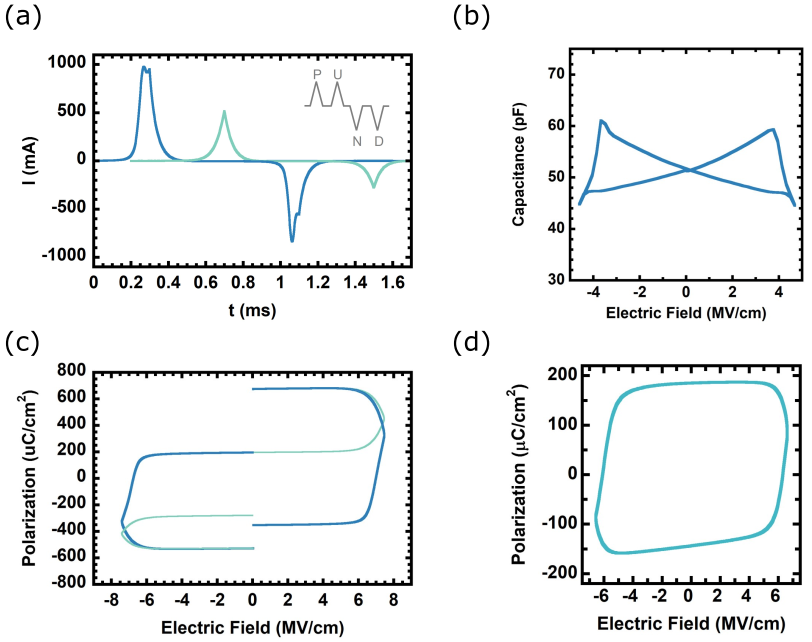

Fig. S8: **PUND and PE measurement** (a) PUND measurement. Blue line is the switched curve (P and N) and light green line shows (U and D) the non-switched curve. Inset denotes the applied voltage triangle pulses with maximum 20V and minimum -20V. (b) Capacitance with respect to the applied electric field. (c) Polarization vs electric field from the triangular pulses PUND measurement. Blue line is the switched curve (P and N) and light green line shows (U and D) the non-switched curve. (d) Polarization vs electric field loop.

Device	Before (cm^2/Vs)	After (cm^2/Vs)
1	42800	43900
2	28200	26900

Table 1: Mobility before and after the exposure

estimation, before the exposure the mobility is around 16400 cm^2/Vs and after the exposure is 11300 cm^2/Vs.

Given the difficulties to compare on this device, to better understand this situation, we have compared mobility from four-terminal measurement before and after exposure in two other devices (Tab. 1).

Previous results from Shi et al. [8] also confirm that well-controlled electron beam exposure does not deteriorate graphene or TMD quality. This confirms that exposing to electron beam under our exposure condition does not deteriorate to the graphene quality.

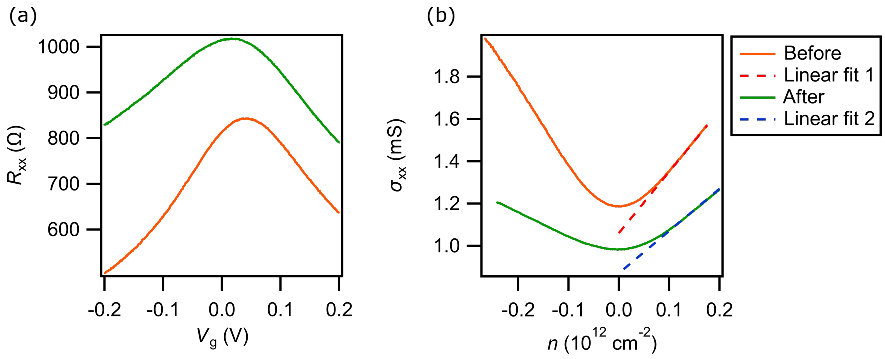

Fig. S9: **Transport measurement** (a) Resistance with respect to the gate voltage before and after the ebeam exposure. (b) Conductivity with respect to the carrier concentration. Linear fit 1 (2) is fitting to get the $\Delta\sigma/\Delta n$ slope of before (after) the exposure.

References

[1] Pierre Hovington, Dominique Drouin, and Raynald Gauvin. Casino: A new monte carlo code in c language for electron beam interaction —part i: Description of the program. *Scanning*, 19(1):1–14, 1997.

[2] Dominique Drouin, Pierre Hovington, and Raynald Gauvin. Casino: A new monte carlo code in c language for electron beam interactions—part ii: Tabulated values of the mott cross section. *Scanning*, 19(1):20–28, 1997.

[3] Pierre Hovington, Dominique Drouin, Raynald Gauvin, David C. Joy, and Neal Evans. Casino: A new monte carlo code in c language for electron beam interactions—part iii: Stopping power at low energies. *Scanning*, 19(1):29–35, 1997.

[4] Jesse Balgley, Jackson Butler, Sananda Biswas, Zhehao Ge, Samuel Lagasse, Takashi Taniguchi, Kenji Watanabe, Matthew Cothrine, David G. Mandrus, Jairo Jr. Velasco, Roser Valentí, and Erik A. Henriksen. Ultrasharp lateral p–n junctions in modulation-doped graphene. *Nano Letters*, 22(10):4124–4130, 2022. PMID: 35533399.

[5] M. M. Fogler, D. S. Novikov, L. I. Glazman, and B. I. Shklovskii. Effect of disorder on a graphene $p-n$ junction. *Phys. Rev. B*, 77:075420, Feb 2008.

[6] Vadim V. Cheianov and Vladimir I. Fal'ko. Selective transmission of dirac electrons and ballistic magnetoresistance of $n-p$ junctions in graphene. *Phys. Rev. B*, 74:041403, Jul 2006.

[7] M. I. Katsnelson, K. S. Novoselov, and A. K. Geim. Chiral tunnelling and the klein paradox in graphene. *Nature Physics*, 2(9):620–625, Sep 2006.

[8] Wu Shi, Salman Kahn, Lili Jiang, Sheng-Yu Wang, Hsin-Zon Tsai, Dillon Wong, Takashi Taniguchi, Kenji Watanabe, Feng Wang, Michael F. Crommie, and Alex Zettl. Reversible writing of high-mobility and high-carrier-density doping patterns in two-dimensional van der waals heterostructures. *Nature Electronics*, 3(2):99–105, Feb 2020.
